# Study of Modified Offset Trajectory for Bonnet Polishing Based on Lifting Bonnet Method

**DOI:** 10.3390/mi14122210

**Published:** 2023-12-06

**Authors:** Shujing Sha, Shaohang Ma, Shanqiang Han, Chenhao Pan, Hang Li, Jieqiong Lin, Mingxing Zhang, Lulu Jiang

**Affiliations:** 1 School of Mechatronic Engineering, Changchun University of Technology, Changchun 130012, China; msh99728@163.com (S.M.); hanshanqiang616@163.com (S.H.); 18746245755@163.com (C.P.); leevoyager19@gmail.com (H.L.); zmxlm@126.com (M.Z.); jllworld97@163.com (L.J.); 2 Key Laboratory of Micro-Nano and Ultra-Precision Manufacturing of Jilin Province, School of Mechatronic Engineering, Changchun University of Technology, Changchun 130012, China

**Keywords:** bonnet polishing, polishing trajectory, lifting bonnet method, modified offset trajectory, edge effect

## Abstract

The inability to converge at the edge of a workpiece during polishing affects the edge profile accuracy and surface quality of the workpiece. In this study, a bias trajectory generation method based on the lifting bonnet method that can maintain the morphology of polished edges is presented. Firstly, by establishing the polishing parameters and the decreasing rule in line with the principles of the lifting bonnet method, we obtained the residual height spacing, the radius of the polishing area, the centre offset position, and the pressing depth for each offset trajectory. Subsequently, the modified bias trajectory algorithm correction coefficients were obtained by fitting the edge trajectories using cubic Bessel curves, which were multiplied with the bias amount to obtain the final modified bias trajectory. Finally, an experiment was designed to compare the edge effect of the modified bias trajectory with the traditional grating trajectory. The experimental findings indicate that the reduction in edge collapse following the implementation of the modified offset trajectory was 1.30 μm. In contrast, the edge collapse after polishing with the traditional grating trajectory amounted to 98.67 μm. Moreover, the edge collapse ensuing traditional polishing trajectory was 75.9 times more pronounced than that observed after using the modified offset trajectory. It is shown that the modified bias trajectory method can not only maintain the original edge morphology of the workpiece but can also promote the convergence of the edge effect to a certain extent.

## 1. Introduction

Ultra-precision polishing technology is frequently applied in the surface treatment of diverse materials, including metals, plastics, ceramics, and silicon carbide [1,2]. This technology serves as a pivotal method for eliminating surface imperfections, enhancing surface finishes, addressing corrective measures, and rectifying sub-surface damage [3]. Bonnet polishing, introduced by D.D. Walker et al. and the London Optical Laboratory, UK, in 2000 [4], employs a bonnet as a polishing tool. It accomplishes grinding and polishing on the workpiece’s surface or the processed surface by inducing abrasive grains to generate a grinding motion through the high-speed rotation of the bonnet’s surface and the rebound pressure exerted during bonnet compression. Notably, an *F*/1.25 parabolic mirror, featuring a diameter of 315 mm, attains a *PV* value of 0.166*λ* (*λ* = 632.8 nm) after polishing with the Zeeko bonnet [5].

In comparison to traditional ultra-precision polishing techniques, bonnet polishing presents several advantages. Firstly, the pressure distribution in the contact region follows an accurate Gaussian-type removal function, contributing to enhanced convergence efficiency [6]. Secondly, the adjustment of pressure within the bonnet enables the creation of a machining tool with a controllable elasticity coefficient, thereby improving the selectivity of machining parameters. Lastly, the convex bonnet exhibits broader applicability due to its overall flexible fit [7]. As the theoretical foundation of bonnet polishing technology advances, research in various domains, including material removal characteristics, process parameter optimization, and trajectory planning, is continuously deepening [8,9]. During the bonnet polishing process, changes in the edge curvature and path may occur, leading to variations in the amount of edge removal on the workpiece. These variations can impact the expected convergence effect on the workpiece’s edge, thereby influencing edge contour accuracy and surface quality [10,11].

The edge effect occurs during polishing when the removal function distorts at the edge due to changes in the force area. This alteration results in variations in the material removal rate at the workpiece’s edge, leading to phenomena such as collapsed or warped edges. To address this issue, Walker et al. [9,12,13] proposed three primary methods for controlling the mirror edge effect: (1) bonding excess material at the workpiece edge; (2) controlling edge flopping during the polishing process and correcting the edge at the end; (3) proactively controlling the edge profile by optimizing polishing parameters. However, bonding excess material may lead to surface deformation and the risk of detachment. The method of controlling edge flopping requires the initial modelling of various sizes of the removal function to accommodate the inclined peripheral edge surface, followed by correcting the edge by finishing with a smaller-sized polishing tool. Walker [14,15] introduced the lifting bonnet method to control the distance from the polishing centre to the edge and the pressing depth of the bonnet. With this method, as the bonnet approaches the edge, the dressing depth decreases until the bonnet is just lifted when it reaches the edge. Simultaneously, the polishing dwell time is adjusted during movement to control the removal amount, reducing the edge effect by avoiding contact with the edge. Additionally, a small pitch tool is used to accommodate the non-spherical mismatched surface of the peripheral region. Li [16] conducted a finite element analysis of the pressure distribution in the edge region to optimize process parameters in the edge area. Based on the Preston equation, this analysis aims to predict the shape of the edge contour. Yu [17] showcased, through a simulation and experiments, that superior edge quality can be achieved by locally rectifying hexagonal optical lenses using small tools compared to machining large surfaces. Others have improved the edge effect by controlling the machining process. For instance, Qu, X. [18] implemented scanning paths and employed numerical methods to determine the optimal spacing value of the polishing paths. They controlled the removal depth by optimizing the feed rate, thus mitigating over-polishing in the edge area. Yin [19] proposed a combined polishing method of double-rotor polishing and spin-polishing to achieve global machining at a constant pressure. The tool influence function (TIF) model to control the edge effect is also a hot research topic among scholars. Hu, H. [20] proposed a heterocercal TIF model, which introduces a specific motion pattern in composite machining equipment to ‘transfer’ the material from the central region ‘to the edges, mainly used in large tool-mirror size ratio conditions. Kim, D.W. [21] introduced a parametric method to represent the spatial distribution of Preston’s coefficients to obtain a parametric fringe TIF model to control the fringe pressure distribution. Jeon, M. [22] combined fringe TIFs with different down-pressure heights and polishing parameters (e.g., step-height of raster scanning paths and speed of movement of the TIFs) and proposed a new mathematical model for predicting fringe effects, and experimentally verified the validity of the proposed model.

The aforementioned efforts have significantly mitigated the edge effect of bonnet polishing. However, there remains ample room for research to enhance the edge effect through trajectory optimization. In this paper, we introduce a polishing offset trajectory generation method based on the lifting bonnet approach, preserving the morphology of the polished edges. The approach involves utilizing offset trajectories with a stepwise decrease in the polishing radius at the edges. This strategy aims to minimize the polishing area leakage at the edges in bonnet polishing and achieve an improved edge effect through the modified offset trajectory. The initial step in the offset trajectory generation method involves applying the lifting bonnet method principle to determine the polishing parameters and decreasing rules. This facilitates the acquisition of crucial information, such as the residual height spacing, polishing area radius, centre offset position, and pressing depth for each offset trajectory. Following this, the algorithm corrects the coefficients by employing a cubic four-node Bezier curve to fit the edge trajectories. The resulting coefficients are then multiplied with the offset amount, yielding the final modified offset trajectory. Lastly, an experiment was devised to compare the edge effect of the modified offset trajectory against the traditional grating trajectory. This experiment aimed to validate the method’s capability to preserve polished edge morphology and ensure effective convergence of the edge effect.

## 2. Offset Trajectory Generation

**Figure 1:**
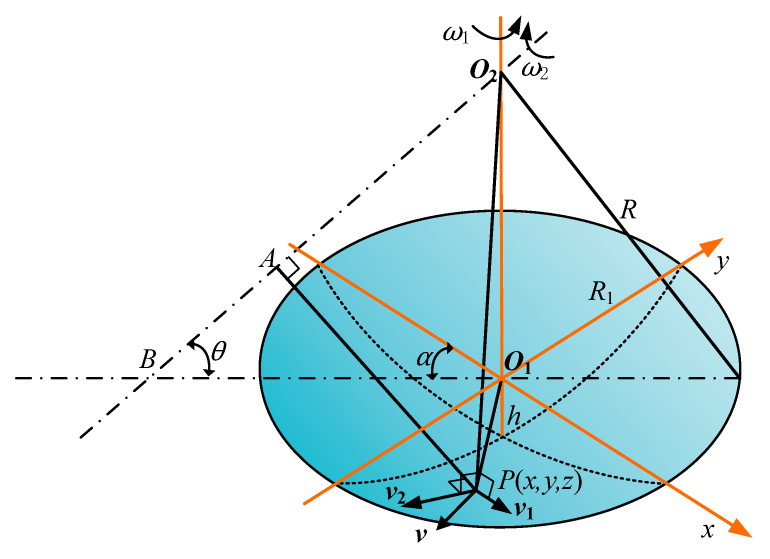
Velocity synthesis schematic for polished point.

For bonnet polishing, Figure 1 illustrates a schematic diagram of the velocity synthesis at an arbitrary processing point *P*. The velocity *v* is composed of two vectors, *v*_1_ and *v*_2_. Here, the bonnet induces velocity *v*_1_ around the rotational axis *O*_1_*O*_2_, while the bonnet contributes to velocity *v*_2_ around the rotational axis *BO*_2_. *ω*_1_ represents the rotational angular velocity, while *ω*_2_ signifies the rotational angular velocity. *R* denotes the radius of the bonnet, and *R*_1_ is the radius of the polishing area. The variable *h* represents the pressing depth on the bonnet, and *θ* denotes the polishing angle of advancement. *O*_1_ is the centre of the processing area, and *O*_2_ is the centre of the bonnet. Point *A* is the vertical foot of the line connecting the two points of *AP* and the rotational axis *BO*_2_. The rotational axis *BO*_2_ intersects with the *xy* plane at point *B*, and *α* is the projected angle of the rotational axis *BO*_2_ in the *xy* plane concerning the *x*-axis.

*v*_1_ is generated as the point *P* rotates around *O*_1_*O*_2_ with an angular velocity of *ω*_1_, while *v*_2_ is generated as the point *P* rotates around *BO*_2_ with an angular velocity of *ω*_2_. The modulus of both velocities can be determined:

(1) $$\;\left\{\begin{array}{c} \;\left|v_{1}\right|{=\omega }_{1}\;\sqrt{{\;x}^{2}{+y}^{2}\;}\\ \left|v_{2}\right|{=\omega }_{2}\;\left|\mathrm{AP}\right| \end{array}\right.\;$$

The direction of *v*_1_ is perpendicular to the plane *O*_1_*O*_2_*P*, and the direction of *v*_2_ is perpendicular to the plane *AO*_2_*P*. These geometric relations yield values for *α*, the coordinates of point *O*_2_, and the coordinates of point *B*:

(2) $$\;\left\{\begin{array}{c} \begin{array}{c} O_{2}\left(0,0,R\;-\;h\right)\\ {\alpha =\omega }_{1}t\\ B\left(\left|{\mathrm{BO}}_{1}\right|\cos\left(\alpha \right),\left|{\mathrm{BO}}_{1}\right|\sin\left(\alpha \right),0\right) \end{array}\\ \left|{\mathrm{BO}}_{1}\right|=\;\left(R\;-\;h\right)/\tan\left(\theta \right) \end{array}\right.$$

The linear equation for two points (*x*_1_, *y*_1_, *z*_1_) and (*x*_2_, *y*_2_, *z*_2_) in 3D space is:

(3) $$\frac{{x\;-\;x}_{1}}{x_{2}{\;-\;x}_{1}}=\frac{{y\;-\;y}_{1}}{y_{2}{\;-\;y}_{1}}=\frac{{z\;-\;z}_{1}}{z_{2\;}{\;-\;z}_{1}}$$

By substituting the coordinates of *B* and *O*_2_ into Equation (3), we obtain the value of *BO*_2_ as follows:

(4) $$\left\{\begin{array}{c} \left(R\;-\;h\right)x+\left|{\mathrm{BO}}_{1}\right|\cos\left(\alpha \right)z=\left|{\mathrm{BO}}_{1}\right|\cos\left(\alpha \right)\left(R\;-\;h\right)\\ \left(R\;-\;h\right)y+\left|{\mathrm{BO}}_{1}\right|\sin\left(\alpha \right)z=\left|{\mathrm{BO}}_{1}\right|\sin\left(\alpha \right)\left(R-\;h\right) \end{array}\right.$$

The equation can be reduced to a matrix in the following form:

(5) $$\left\{\begin{array}{c} \mathrm{eu}=b\\ e=\left(\begin{array}{ccc} 1 & 0 & \frac{\cos\left(\alpha \right)}{\tan\left(\theta \right)}\\ 0 & 1 & \frac{\sin\left(\alpha \right)}{\tan\left(\theta \right)} \end{array}\right)\\ b=\left(\begin{array}{c} \frac{\left(R\;-\;h\right)\cos\left(\alpha \right)}{\tan\left(\theta \right)}\\ \frac{\left(R\;-\;h\right)\sin\left(\alpha \right)}{\tan\left(\theta \right)} \end{array}\right)\\ u={\left(x,y,z\right)}^{T} \end{array}\right.$$

where *u* represents any point on the line. Bonnet polishing is a precision polishing process with minimal material removal both before and after polishing. Considering the limited impact of material removal on speed, it is assumed that the plane height remains constant over the polishing time in the derivation of the equation. If the coordinates of point *P* in the polishing area are represented as *p* = (*x*, *y*, *z*)^T^, then the length of *AP* is:

(6) $$\left|\mathrm{AP}\right|=\sqrt{{\left(b\;-\;e\;\times \;p\right)}^{T}{\left({\mathrm{ee}}^{T}\right)}^{-1}\left(b\;-\;e\;\times \;p\right)}$$

Since the influence of *v*_1_ on the final velocity *v_p_* is negligible, the velocity function can be simplified at time *t*:

(7) $$\;V(x,y,z,t)=\left|v_{2}\right|{=\omega }_{2}\;\times \;\left|\mathrm{AP}\right|\;$$

Taking into account the circular shape of the bonnet polishing area, the pressure *P* at any point within the contact zone can be calculated as:

(8) $$\;P\left(x,y,z\right)=\frac{F}{\pi h\left(2R\;-\;h\right)}=-\frac{k_{c}\left(\sqrt{R^{2}\;{-\;x}^{2}\;{-\;y}^{2}}\;-\;R+h\right)}{\pi \left({2\mathrm{Rh}\;-\;h}^{2}\right)}\;$$

where *k_c_* is the elasticity coefficient of the bonnet, *F* is the contact force on the workpiece, and the point of maximum average pressure occurs when *r* = 0, *x* = 0, *y* = 0, *z* = 0:

(9) $$\;P_{\max}\left(x,y,z\right)=\frac{k_{c}}{\pi \left(2R\;-\;h\right)}\;$$

The minimum point of average pressure on the edge points is:

(10) $${\;P}_{\min}\left(x,y,z\right)=0\;$$

The equation for the removal function is derived from Equation (7) for velocity and Equation (8) for pressure and is expressed as:

(11) $$\frac{\mathrm{dH}\left(x,y,z\right)}{\mathrm{dt}}=\mathrm{KV}\left(x,y,z\right)P\left(x,y,z\right)$$

where *K* is the scale factor. The equation can be simplified to:

(12) $$\;H\left(x,y,z\right){=K\omega }_{2}\sqrt{{\left(b\;-\;A\;\times \;p\right)}^{T}{\left({\mathrm{AA}}^{T}\right)}^{-1}\left(b\;-\;A\;\times \;p\right)}\;\frac{k_{c}\left(-\sqrt{R^{2}\;{-\;x}^{2\;}{-\;y}^{2}}+R\;-\;h\right)}{\pi \left({2\mathrm{Rh}\;-\;h}^{2}\right)}t\;$$

**Figure 2:**
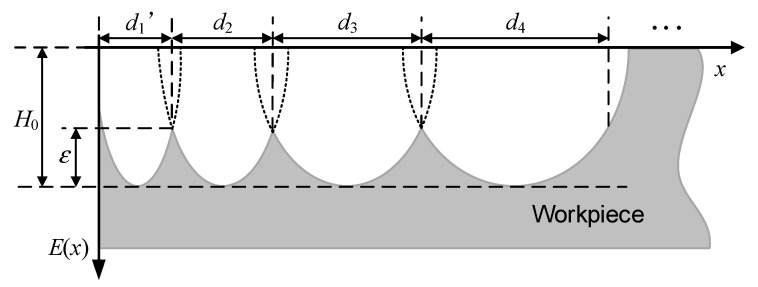
Schematic of controlling edge row spacing to maintain the equal residual height.

The purpose of the modified offset trajectory for bonnet polishing, based on planar planning, is to utilize the offset trajectory method to reduce the missed polishing area at the edge of the bonnet, thereby achieving a modified edge effect. As indicated in the initial section introducing the lifting bonnet method, the method stipulates that the pressing depth should decrease as the polishing region approaches the edge. Consequently, resolving the line spacing control for the offset trajectory becomes particularly crucial. A schematic representation of edge row spacing control under equal residual heights is illustrated in Figure 2. Here, *d_n_* represents the offset distance for equal residual heights (labelled *d*_1_′ to distinguish it from *d*_1_, which is more specific). *ε* denotes the residual height, *E*(*x*) describes the variation rule of the residual heights *ε* and *R*_1_, and *H*_0_ represents the depth of removal from the centre point of the polished area.

The depth of the workpiece after polishing is calculated by using the speed of the centre point in the polishing area instead of the speed of each point in the polishing area. Substituting *y* = 0 and *z* = 0 into Equation (12) yields the following equation:

(13) $$E(x)\;=\;Kk_{c}\omega_{2}(R\;-\;h)\cos\theta \frac{\;-\sqrt{R^{2}\;{-\;x}^{2}}+R\;-\;h}{{\pi (2\mathrm{Rh}\;-\;h}^{2})}t\;$$

Equation (13) is in the form of the pressing depth, which is transformed into the form of the radius of the polishing area as follows:

(14) $$\;E\left(x\right)=t\frac{{\mathrm{Kk}}_{c}\omega_{2}\cos\theta }{{\pi R}_{1}^{2}}\left(R^{2}\;{-\;R}_{1\;}^{2}-\;\sqrt{{(R}^{2}{-\;R}_{1}^{2}{)(R}^{2}\;{-\;x}^{2})}\right)\;$$

Setting the polishing depth *E*(*x*) = *ε* − *H*_0_ and substituting it into Equation (14) obtains:

(15) $${\;\varepsilon \;-\;H}_{0}=t\frac{{\mathrm{Kk}}_{c}\omega_{2}\cos\theta }{{\pi R}_{1}^{2}}\left(R^{2}\;{-\;R}_{1\;}^{2}-\;\sqrt{{(R}^{2}\;{-\;R}_{1}^{2}{)(R}^{2}\;{-\;x}^{2})}\right)\;$$

In Equation (15), *R*_1_ is the radius of the polishing area, and *R*_1*n*_ is the radius of the *n*th polishing area. The radius of the offset polished area, *R*_1*i*_, for the *i*th, offset iteration can be determined by applying the decreasing rule, where *i* ∈ (2, *n*]. The dwell time *t_i_* and the offset distance *d_n_* for equal residual height on each trajectory can be determined by solving constraint Equation (15). Based on the geometrical relationship between the pressing depth and the polishing area, the corresponding pressing depth *h_i_* can be obtained, resulting in the final offset position *p* of the trajectory’s centre.

(16) $$\left\{\begin{array}{c} p_{1}{=R}_{11}\\ {\;p}_{i}{=p}_{i-1}{+(d}_{i-1}{+d}_{i})\;/\;2 \end{array}\right.\;,\;i\in (2,\left.n\right]$$

The equation, derived by applying the geometric progression decreasing rule to modulate the change in the radius of the bonnet polishing area by dividing it with the common ratio *q*, is outlined below:

(17) $$\frac{R_{11}}{q^{0}}=\frac{R_{12}}{q^{1}}=\frac{R_{13}}{q^{2}}=\cdots =\frac{R_{1n-3}}{q^{n-3}}=\frac{R_{1n-2}}{q^{n-2}}=\frac{R_{1n-1}}{q^{n-1}}$$

**Figure 3:**
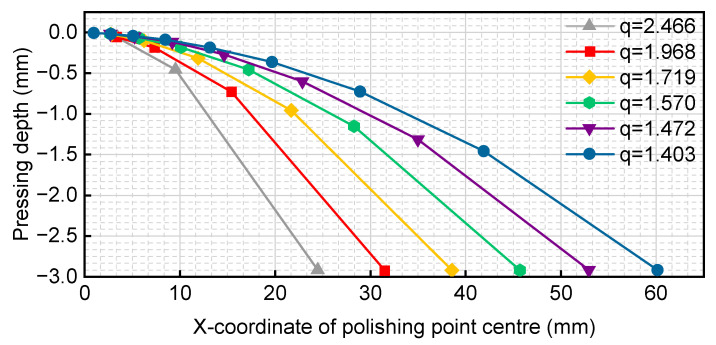
Variation curve of pressing depth with position of polishing centre.

We substituted the simulation experiment data from Table 1 into Equation (15). In Table 1, *L* represents the edge row spacing control coefficient, denoting the maximum control offset trajectory row spacing. The radius of the controlled bonnet polishing area varies from 0 to 15 mm. The resulting values for the residual height spacing *d*, radius of the polishing area *R*_1_, centre offset position *p*, and pressing depth *h* are presented in Table 2:

The pressure versus polishing centre curve is depicted in Figure 3. Each curve in Figure 3 corresponds to a radius of 15 mm. The horizontal axis represents the location of the centre of the polishing area on the workpiece, while the vertical axis represents the pressing depth of the bonnet. The pressing depth does not vary linearly based on its position. As the initial bonnet indentation radius, *R*_1_ is a fixed value when *q*^0^ = 1, an edge scaling coefficient can be established. The offset obtained through the geometric ratio method is then multiplied by this coefficient, resulting in the edge row spacing control coefficient *L* being divided by the edge scaling coefficient.

The modified algorithm initially requires fitting the edge points into a form that can be represented by a mathematical function. The Bezier curve is defined by a start point, an end point, and two control points, each with definite coordinates, making the position of the Bezier curve in space very clear. Furthermore, the shape of the Bezier curve is determined by the position of the control points, making it highly responsive to changes in shape. Since this paper requires specific start and end points, the target curve is approximated using cubic Bezier curves segments [23,24,25], and the data points are approximated through least squares fitting.

The single Bezier curve is defined as:

(18) $$\;q\left(t_{i}\right)=\overset{m}{\underset{k=0}{\sum }}\frac{m!}{k!\left(m\;-\;k\right)!}P_{k}{\left({1\;-\;t}_{i}\right)}^{m-k}t_{i}{}^{k}\;,\;0\;\leq \;t\;\leq \;1\;$$

where *q*(*t_i_*) represents the interpolation point at the parameter value *t_i_*, *m* is the order of the Bezier curve, and *P_k_* is the *k*th node. The segmental approximation of the target curve using a cubic four-node Bezier curve is given by the following equation:

(19) $$\;q\left(t_{i}\right)=\;{\left({1\;-\;t}_{i}\right)}^{3}P_{0}{+3t}_{i}{\left({1\;-\;t}_{i}\right)}^{2}P_{1}{+3t}_{i}{}^{2}\left({1\;-\;t}_{i}\right)P_{2}{+t}_{i}{}^{3}P_{3}\;$$

The shape of the cubic Bezier curve is determined by the position of the control points, where *P*_0_ and *P*_3_ represent the first and last nodes, and *P*_1_ and *P*_2_ correspond to the middle nodes, as shown in Equation (19). The optimal intermediate node position is determined by adjusting *P*_1_ and *P*_2_ to calculate the fit error, minimising the squared distance between the initial and fitted data using the least squares method. Assuming there are *n* trajectory data points that need to be fitted, and *p_i_* and *q*(*t_i_*) represent the original and approximation values respectively, the least squares equation can be expressed as follows:

(20) $$\;S=\overset{n}{\underset{i=1}{\sum }}{\left[p_{i}\;-\;q\left(t_{i}\right)\right]}^{2}\;$$

After establishing the initial set of endpoints as the start and end of the trajectory, *P*_1_ and *P*_2_ are determined using Equation (21).

(21) $$\frac{\partial S}{\partial P_{1}}=\frac{\partial S}{\partial P_{2}}=0$$

This equation is solved to obtain:

(22) $$\;P_{1}=\frac{A_{2}C_{1}\;{-\;A}_{12}C_{2}}{A_{1}A_{2}\;{-\;A}_{12}A_{12}}\;$$

(23) $$\;P_{2}=\frac{A_{2}C_{2}\;{-\;A}_{12}C_{1}}{A_{1}A_{2}\;{-\;A}_{12}A_{12}}\;$$

(24) $${\;A}_{1}=9\overset{n}{\underset{i=1}{\sum }}t_{i}{}^{2}{\left({1\;-\;t}_{i}\right)}^{4}\;$$

(25) $${\;A}_{2}=9\overset{n}{\underset{i=1}{\sum }}t_{i}{}^{4}{\left({1\;-\;t}_{i}\right)}^{2}\;$$

(26) $${\;A}_{12}=9\overset{n}{\underset{i=1}{\sum }}t_{i}{}^{3}{\left({1\;-\;t}_{i}\right)}^{3}\;$$

(27) $$\;C_{1}=\overset{n}{\underset{i=1}{\sum }}{3t}_{i}{\left({1\;-\;t}_{i}\right)}^{2}\left[p_{i}\;-\;{\left({1\;-\;t}_{i}\right)}^{3}P_{0}\;{-\;t}_{i}{}^{3}P_{3}\right]\;$$

(28) $${\;C}_{2}=\overset{n}{\underset{i=1}{\sum }}{3t}_{i}{}^{2}\left({1\;-\;t}_{i}\right)\left[p_{i}\;-\;{\left({1\;-\;t}_{i}\right)}^{3}P_{0}\;{-\;t}_{i}{}^{3}P_{3}\right]\;$$

**Figure 4:**
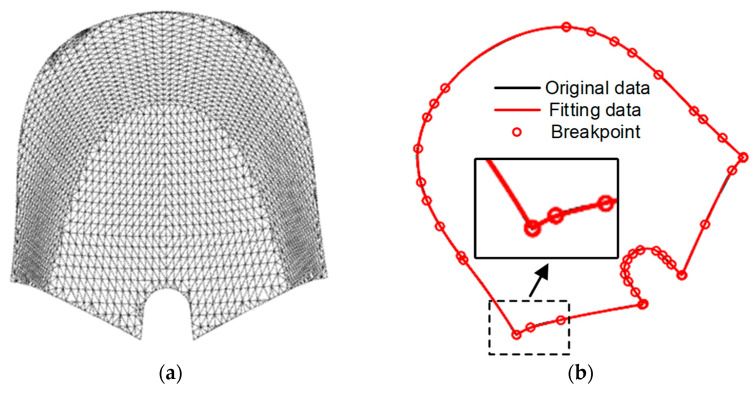
Edge fitting effect diagram: (**a**) Grid map of the original contour to be fitted; (**b**) fitting edge contour plots.

Due to the limited flexibility of the cubic four-node Bezier curves in fitting complex trajectories, when a significant error exists between the fitted trajectory data and the original trajectory data, it becomes necessary to subdivide line segments that do not meet the error criterion. This involves splitting the segments from the point of maximum error into two segments and re-fitting them again to control the whole trajectory error within the error limit. Taking the surface of a branded mouse as an example, the original grid is illustrated in Figure 4a. Setting the maximum error between the fitted trajectory data and the original trajectory data to 0.001, Figure 4b depicts the results, where the black line represents the original trajectory, and the red line represents the fitted trajectory. The black line is completely covered by the red line, resulting in a fitting error of 9.534 × 10^−4^, as calculated by the algorithm mentioned above. The obtained result aligns well with the intended purpose.

**Figure 5:**
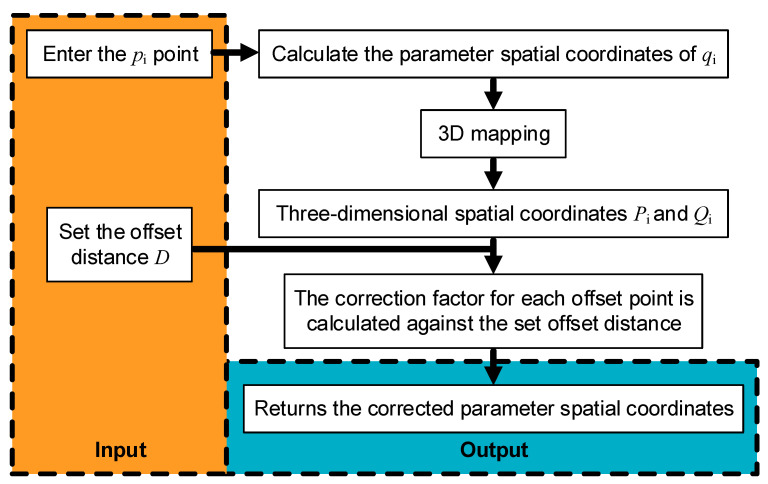
Flowchart of the algorithm for solving each corrective factor.

The process of solving the corrected coefficients for the modified offset trajectory algorithm is illustrated in Figure 5.

The modified offset trajectory algorithm is described as follows:

1. The whole surface offset trajectory is divided into edge and interior parts, and the edge spacing is obtained based on the above. The internal offset is set to the value of the last digit of the edge spacing;
2. Apply the cubic Bezier curve to accurately fit the curve, establish the bonnet direction, and compute the normal;
3. Based on the current number of offsets in the edge row spacing value at position index *D_max_*, the curve fitted in step 2 will experience an offset of *D_max_*, resulting in the offset curve. The curve before the offset is referred to as *p*, while the curve after offset is known as *q*. Discretise the curve *p* as a sequence ${\{p}_{i},\;i=0,1\ldots ,\;n\}$, The curve *q* is discretised as a sequence ${\{q}_{i},\;i=0,1\ldots ,\;n\}$;
4. Mapping *p_i_* and *q_i_* to a spatial surface, we obtain ${\{P}_{i}{,\;i=0,1\ldots ,\;n\},\;\{Q}_{i},\;i=0,1\ldots ,\;n\}$;
5. First, calculate the distance *d_i_* between each *p_i_* and *q_i_* offset combination. Next, calculate the distance *D_i_* and deviation $\varepsilon_{i}{=(D}_{i\;}{-\;D}_{\max}{)\;/\;D}_{i}$ between the offset pairs *P_i_* and *Q_i_*. Finally, correct *d_i_* to $d_{i}{\prime =1\;-\;\varepsilon }_{i}$ and adjust *q_i_* according to *d_i_′* as ${\{q}_{i}\prime ,\;i=0,1\ldots ,\;n\}$;
6. Detect whether the curve completely covers the parameter plane. Cease repetition if it does, otherwise proceed to step 2;
7. Output all offset trajectories.

## 3. Experimental Polishing Verification

**Figure 6:**
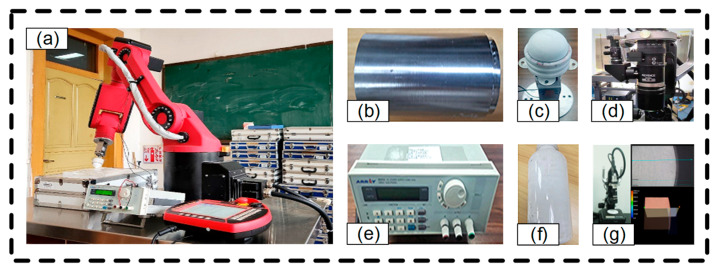
Diagram of the equipment needed for conducting bonnet polishing experiments: (**a**) Industrial robot; (**b**) Experimental workpiece; (**c**) Bonnet polishing head; (**d**) Lens; (**e**) Current Source; (**f**) Polishing fluid; (**g**) Detection Instruments.

The verification experiment was conducted on the bonnet polishing platform of an industrial robot, utilizing a spherical ethylene–vinyl acetate copolymer (EVA) polishing head and ceria polishing solution. The surface quality and contour of the machined surface were acquired using the Keyence VHX-5000 (KEYENCE, Osaka, Japan) optical measuring instrument. The structure of the experimental setup and the necessary instruments are illustrated in Figure 6.

The bonnet diameter used in the experiment was 40 mm, and the speed range of the bonnet was 0 to 500 rpm. The models and parameters of the experimental apparatus and equipment are detailed in Table 3.

For this paper, the selected workpiece for machining was a circular plane of an aluminium alloy mould with a diameter of 49 mm. The workpiece being processed exhibited visible scratches. To estimate the convergence value of the edge effect, a convergence experimental study was conducted using the contour line without polishing as the reference for convergence. To assess the edge effect of the modified offset trajectory during actual machining, the conventional grating trajectory was employed as the reference object for a comparative analysis of convergence between the two methods.

**Figure 7:**
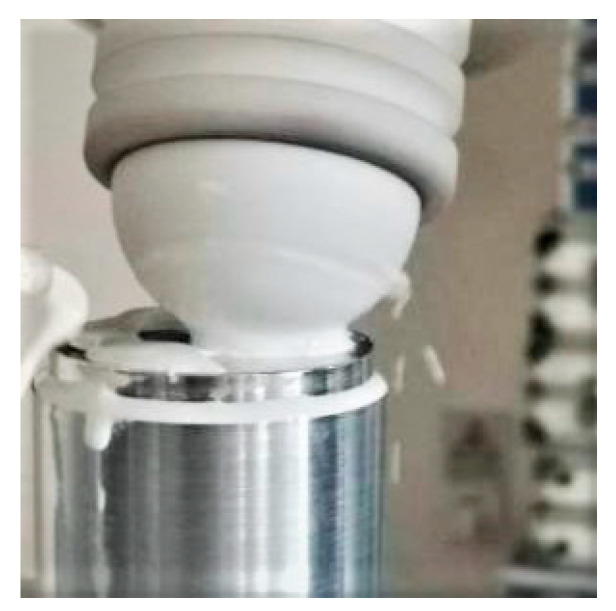
Workpiece polishing experimental process.

For comparison, the polished surface was divided into two semicircular polishing areas that were symmetrical to the left and right sides. On one side, the processing trajectory followed the modified offset trajectory, while on the other side, it followed the conventional grating trajectory. The fixed workpiece controlled the industrial robot polishing platform for polishing, and the polishing process is depicted in Figure 7.

The polishing process necessitates the initial setup of the offset trajectory. After undergoing five offset iterations, the state of geometric progression offset was attained. Subsequently, the edge scaling factor was set to 0.1, and *d* = 1.8 *r* (where each step of *d* in the trajectory was 0.9 times the diameter of the polishing area). After determining the radius of the offset polishing area using Equation (17), the single-point dwell time for each trajectory at the removal depth at the centre of the uniformly polished area was calculated by substituting it into Equation (15). Subsequently, the pressing depth for each trajectory was derived based on the geometric relationship between the pressing depth and the polishing area outlined in Equation (13). Finally, the offset position for the centre of each trajectory was obtained, as summarized in Table 4.

**Figure 8:**
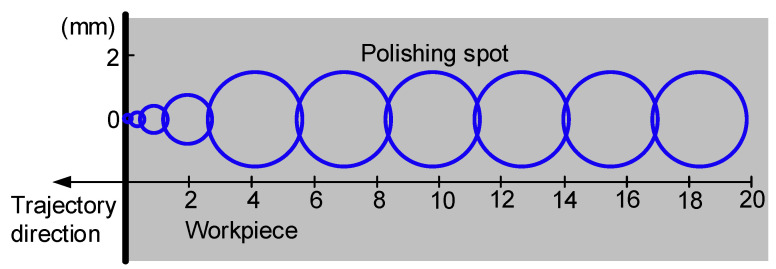
Arrangement of polished spots from the edge (left side) to the centre (right side).

To observe the polishing radius and the relationship between the bonnet location, the distribution, and the arrangement of the workpiece radius determine the size and location of the polished spot, as illustrated in Figure 8. The left side of the diagram represents the polishing edge, and the right side depicts the workpiece centre. The polished spot becomes smaller the closer the half warp is to the edge, in accordance with the lifting bonnet method.

**Figure 9:**
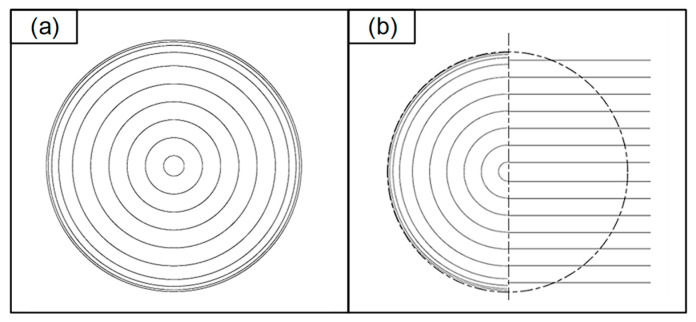
Splices of the trajectories employed in the edge effect experiments: (**a**) Modified offset trajectory used for the test; (**b**) comparative experiment of two trajectories.

**Figure 10:**
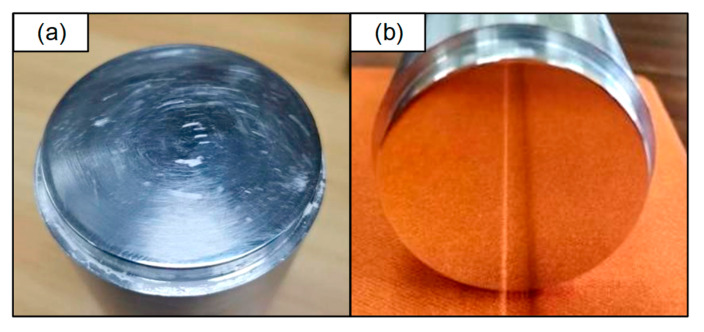
Before and after the workpiece polishing effect: (**a**) Workpiece effect before polishing; (**b**) workpiece effect after polishing.

The traditional grating trajectory set in the experiment has a pitch of 0.292 mm and a single-point dwell time of 301.630 s. To facilitate the comparison of edge effect convergence, the right half of the workpiece is processed using the traditional grating trajectory. The modified offset trajectory and the traditional grating trajectory are then spliced and aligned to obtain the comparison of the experimental processing trajectory, as illustrated in Figure 9. In Figure 9b, the left side shows the modified offset trajectory, while the right side depicts the traditional grating trajectory used as the control. The polishing process lasted for three hours, and the before-and-after polishing effects on the workpiece are presented in Figure 10.

**Figure 11:**
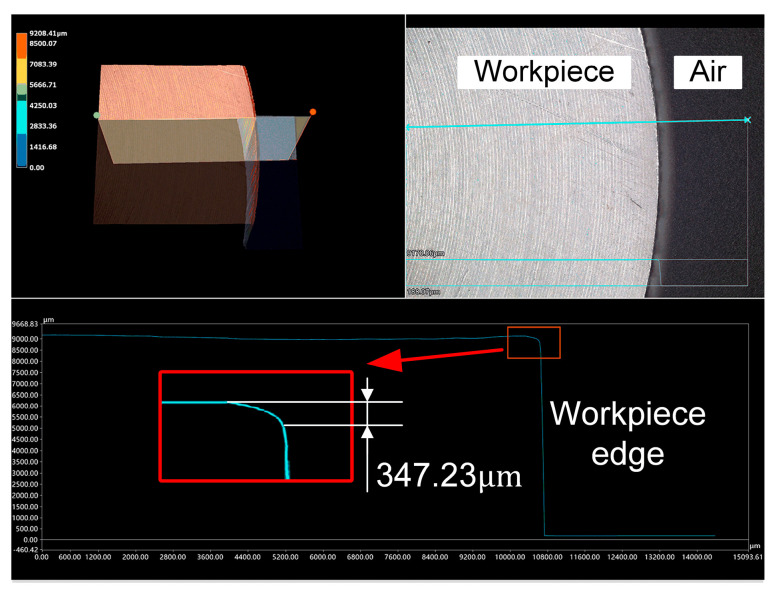
Edge profile of the workpiece before polishing.

**Figure 12:**
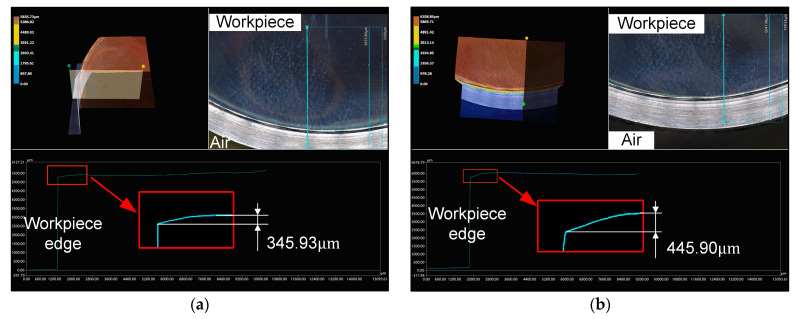
Edge profile of the workpiece: (**a**) Workpiece edge profile after polishing with modified offset trajectory; (**b**) workpiece edge profile after polishing with conventional grating trajectory.

The edge contour lines of the workpiece surface before and after machining were measured using the VHX-5000 (KEYENCE, Osaka, Japan) digital microscope, as illustrated in Figure 11 and Figure 12. The red square area in Figure 11 highlights the collapsed edge of the original workpiece. Figure 12a displays the edge contour of the workpiece after polishing with the modified offset trajectory, while Figure 12b depicts the edge contour of the workpiece after polishing with the conventional grating trajectory.

To accurately depict the edge profile processing effect of bonnet polishing using the modified offset trajectory, by measuring the longitudinal distance from the highest point of the edge profile curve to the edge position of the workpiece in Figure 11 and Figure 12, we determined the original edge profile collapse amount, the edge profile collapse amount after the modified offset trajectory processing and after the traditional grating trajectory processing. The obtained measurements are presented in Table 5.

Upon comparing the measured data in Table 5, it can be observed that the amount of edge collapse after polishing with the modified offset trajectory (345.93 μm) was essentially the same as the amount of edge collapse before polishing (347.23 μm). This indicates that the modified offset trajectory maintained the original edge morphology of the polished workpiece. In contrast, the amount of edge collapse change after polishing with the traditional polishing trajectory was 75.9 times greater than the amount of edge collapse change after polishing with the modified offset trajectory. This suggests that the offset trajectory can promote the convergence of the edge effect to a certain extent, and the convergence effect was superior to that of the traditional grating trajectory.

## 4. Discussion of Relevant Conditions

### 4.1. Comparison of Edge Effects between Traditional Lifting Bonnet Method Trajectories and Modified Offset Trajectories

**Figure 13:**
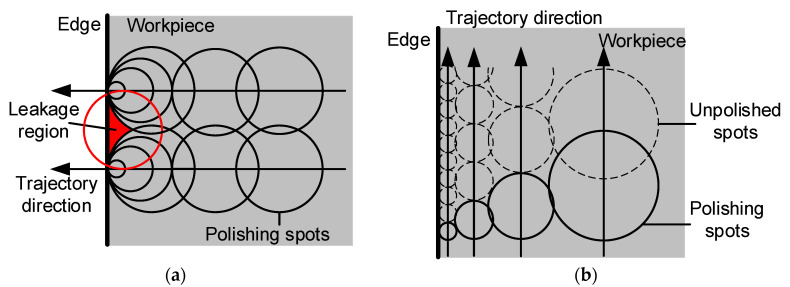
Schematic diagram of the trajectory of the lifting bonnet method: (**a**) Trajectory in the direction of the vertical edge; (**b**) trajectories in the direction of the parallel edge.

For planar trajectory planning, various trajectories are available, including grating trajectories, concentric circle trajectories, spiral trajectories, pseudo-random trajectories, and isometric offset trajectories [26,27,28]. Grating and pseudo-random trajectories generate paths perpendicular to some edges, resulting in the creation of many leakage regions at the edges. The selection of a polishing trajectory for the lifting bonnet method should consider edge effects. As illustrated in Figure 13a, the red region of the red circle remains partially unprocessed. Taking the trajectory direction shown in Figure 13b, where the trajectory direction aligns with the edge direction of the bonnet trajectory planning method, can help reduce the occurrence of leakage regions.

**Figure 14:**
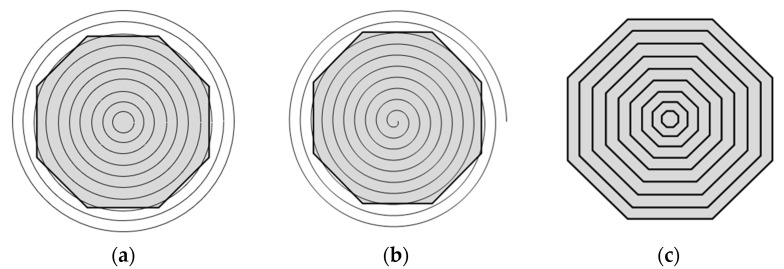
Trajectory schematic: (**a**) Concentric circle trajectory; (**b**) spiral trajectory; (**c**) isometric offset trajectory.

As depicted in Figure 14, while trajectories generated by concentric circle and spiral trajectories are not perpendicular to the surface edge, they both exist at a certain angle to the edge. The isometric offset trajectory, on the other hand, meets all the requirements. It not only maintains the trajectory parallel to the edge, as shown in Figure 14b but also ensures complete coverage of the surface. From a tangent direction perspective, the edge effect of the isometric offset trajectory is superior to that of concentric circle and spiral trajectories.

**Figure 15:**
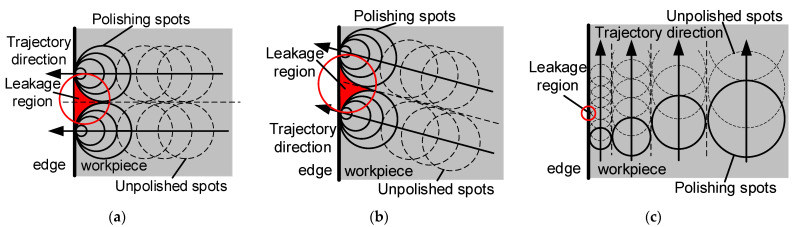
Three forms of edge processing for the lifting bonnet method: (**a**) Perpendicular cuts; (**b**) oblique edge cuts; (**c**) parallel cuts.

In terms of operational complexity and trajectory selectivity, the efficiency of using the lifting bonnet method surpasses that of the edge extension method and the approach angle optimization method. The forms of edge processing in the lifting bonnet method, as illustrated in Figure 15, are categorized into three forms based on the relationship between the trajectory and the edge: perpendicular cuts, oblique edge cuts, and parallel cuts.

**Figure 16:**
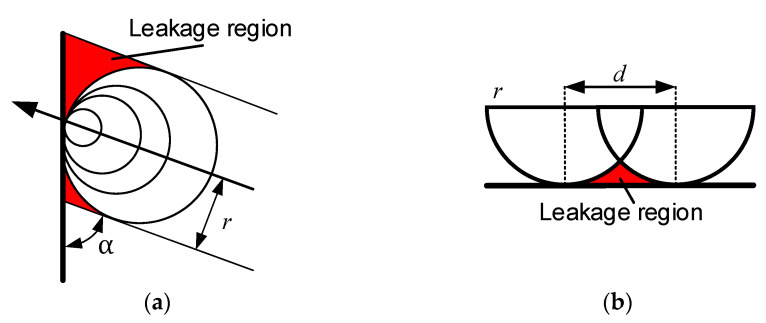
Schematic of unpolished areas: (**a**) oblique edge cut-in; (**b**) parallel edge cut-in.

The two forms of perpendicular cuts and oblique edge cuts, as illustrated in Figure 15a,b, can be categorized as non-parallel machining forms. In each toolpath, two red residual areas are observed, as depicted in Figure 16a.

The direction of the arrow indicates the cutting direction of the polishing trajectory, and the residual area *S*_1_ at the edge of the red area in Figure 16 can be determined from the deflection angle *α* and the radius of the polishing area *r*:

(29) $$\;S_{1}{=r}^{2}\left(\frac{1}{\tan\left(\frac{\alpha }{2}\right)}-\frac{1}{\tan\left(\frac{\alpha \;-\;\pi }{2}\right)}+\frac{\pi }{2}\right)\;$$

As illustrated in Figure 16b, the residual area *S*_1_ in the case of parallel edges can be derived from the step size *d* and the radius of the polishing area *r*, where *r* ≤ *d* ≤ 2*r*.

(30) $${\;S}_{2}=\mathrm{rd}\;-\;\frac{{\pi r}^{2}}{2}+\left(\mathrm{acos}\left(\frac{d}{2r}\right)r\;-\;\frac{1}{2}\sqrt{r^{2}-\frac{d^{2}}{4}}\right)d\;$$

Equations (29) and (30) were implemented in MATLAB (9.6.0.1072779, R2019a) to calculate the edge residual area of the lifting bonnet method in non-parallel and parallel classes at the edge. To eliminate the influence of shape on the trajectory analysis, a rectangle was used to simulate the edge, with one edge serving as a closed edge and the remaining three edges open, constructing a single-edge open machining area for computational validation. The calculated edge residual area is presented in Table 6. From the analysis in Table 6, it can be observed that the smaller the trajectory cut-in direction, the larger the residual area of unpolished area caused by the use of the lifting bonnet method. When the cutting direction was perpendicular to the edge, the non-parallel class lifting bonnet method exhibited the smallest total edge residual area.

Under the same conditions, testing the parallel class trajectories yields the results of the edge residual area, as shown in Table 7.

As evident from Table 7, the smaller the step size of the parallel class of the lifting bonnet method, the smaller the total edge residual area of the trajectory at the edge. When *d* = 2*r* is reached, the effect is similar to the best result for the non-parallel class. It can be concluded that the quality of the edge effect is influenced by the relationship between the trajectory and the edge. The lifting bonnet method offset trajectories exhibit superior edge effects compared to other trajectories.

### 4.2. Comparison of Offset Distances for the Decreasing Rule for Geometric Progression and Arithmetic Progression

The polished area’s radius increases gradually from the edges towards the interior. If the residual height spacing *d* is too small, it causes an excessively long machining trajectory. Create the arithmetic progression decreasing rule with tolerance *R*_11_.

(31) $$\frac{R_{11}}{1}=\frac{R_{12}}{2}=\frac{R_{13}}{3}=\cdots =\frac{R_{1n-2}}{n\;-\;2}=\frac{R_{1n-1}}{n\;-\;1}=\frac{R_{1n}}{n}$$

Setting the residual height *ε* to 0.050 mm, the same isotropic series of simulation experimental data is substituted into the constraints, and the centre removal depth *H*_0_ is calculated using Equation (15). The control of the bonnet polishing radius *R*_1_ varies within the range of 0~15 mm in an isotropic series. The obtained polishing area radius *R*_1_, residual height spacing *d*, centre offset position *p*, and pressing depth *h* are presented in Table 8.

**Figure 17:**
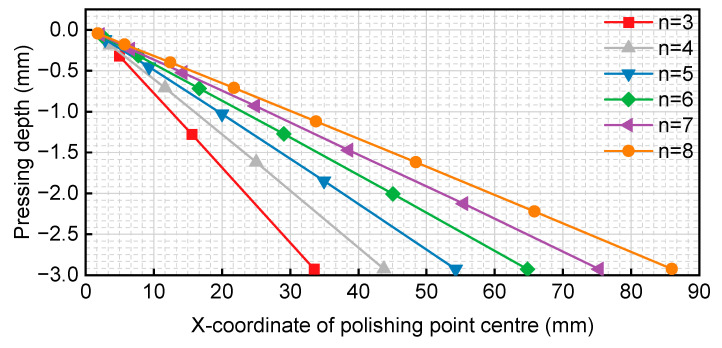
Variation curves of pressing depth with the position of polishing centre.

As indicated in Table 8, *d*, *p*, and *h* increase with the increasing polishing area radius *R*_1_, and the variations in edge row spacing align with the expectations of the control design. Since the pressing depth is a linear function of the offset distance and the radius of the polishing area at each step, a more intuitive relationship between pressing depth and position was selected for display. Keeping the remaining conditions unchanged, the pressing depth was calculated for multiple sets of *n* values along with the polishing centre position curves, as illustrated in Figure 17.

The radius of the polishing area of the bonnet for each curve in Figure 17 follows an arithmetic progression ranging from 0 to 15 mm, with the number of marked points denoted as *n*. The horizontal axis represents the position of the centre point of the polishing area on the workpiece, while the vertical axis represents the pressing depth of the bonnet. From Figure 17, it can be concluded that, while maintaining an equal residual height and removal depth, the larger the value of *n*, the larger the transition area of polishing. For a single curve, the amount of pressing depth to be adjusted is linearly related to the position.

**Figure 18:**
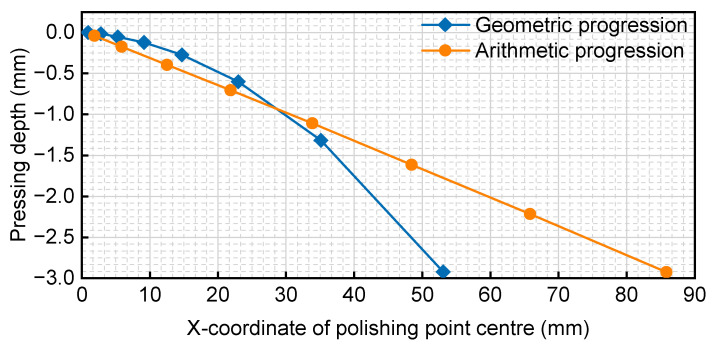
Comparison of positioning and pressing depth of two methods for *n* = 8.

**Figure 19:**
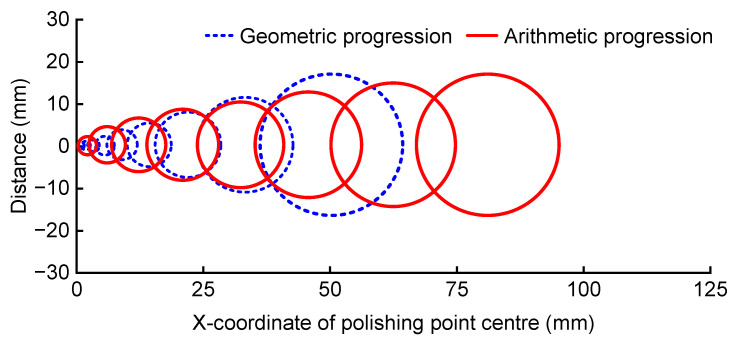
Comparison of the position and radius of the polishing area for two paths in the case of *n* = 8.

The two methods of geometric progression and arithmetic progression were compared using the same value of *n* (*n* = 8), and the relationship between the coordinates of the polished centre and the pressing depth is illustrated in Figure 18 and Figure 19.

The blue (dashed) and red (solid) lines represent the geometric progression decreasing method and the arithmetic progression decreasing method, respectively. From the earlier analysis of the lifting bonnet method, it is evident that the bonnet needs to be just out of contact at the edge. In the comparative analysis of the side close to the edge, it was observed that the contact area range of the geometric progression decreasing method was smaller than that of the arithmetic progression decreasing method, allowing the radius of the polishing area to vary quickly towards the set value. The arithmetic progression decreasing method exhibited a more uniform and slower change. Considering these factors, the method of generating the offset distance with the decreasing rule of the geometric progression was chosen.

## 5. Conclusions

We introduced a modified offset trajectory based on the lifting bonnet method for trajectory planning on polished surfaces and validated its effectiveness in enhancing edge effects. To ensure the convergence of the edge effect on a polished workpiece, we determined the residual height spacing, the radius of the polished area, the centre offset position, and pressing depth by setting the polishing parameters and decreasing rules for the offset trajectory generation method in accordance with the principles of the lifting bonnet method. Subsequently, the correction coefficients for the modified offset trajectory algorithm were determined by fitting the edge trajectory using a cubic four-node Bezier curves. These coefficients were then multiplied with the offset amount to obtain the final offset trajectory. Finally, an experiment comparing the edge effect of the modified offset trajectory with the traditional grating trajectory was designed and verified by polishing the surface of the workpiece using an industrial robotic bonnet polishing platform.

The experimental results reveal that the collapsed edge change after utilizing the modified offset trajectory was 1.30 μm, whereas the collapsed edge change after polishing with the traditional grating trajectory was 98.67 μm. The collapsed edge change after polishing with the traditional polishing trajectory was 75.9 times that of the collapsed edge change after polishing with the modified offset trajectory. This demonstrates that the modified offset trajectory method can not only preserve the original edge morphology of the workpiece but can also promote the convergence of the edge effect to a certain extent.

Furthermore, with the cubic Bezier curves, we fitted the model edges and conducted the offset trajectory through the normal of the fitted curves. This approach calculates the offset correction coefficient based on the difference between the offset distance and the set offset distance. The offset correction coefficient is then used to correct the offset distance of each fitted point, completing the modified offset trajectory operation. In the calculation of this correction coefficient, the cubic four-node Bezier curves may need to split the line segments when the error between the fitted trajectory data and the original trajectory data is too large. For more intricate polished trajectories, we will explore complex curve fitting methods, such as B-spline curves, and these considerations merit further discussion.

## Figures and Tables

**Table 1:** Simulation experimental data.

Parametric	Numerical Value
Bonnet radius	40 mm
Angle of advance	π/6 rad
Self-transmitted angular velocity *ω*_2_	25 r/s
Residual height spacing *d*	Geometric progression
Edge row spacing control coefficient *L*	15
Number of edge-filling trajectories *n*	*n*
Combined coefficient *m*	0.001

**Table 2:** Simulation experimental results of the geometric progression decreasing rule.

Radius of Polishing Area *R*_1_ (mm)	Residual Height Spacing *d* (mm)	Centre Offset Position *p* (mm)	Dwell Time *t* (s)	Pressing Depth *h* (mm)
1.000	1.414	1.000	29.025	0.013
1.472	2.082	2.748	29.030	0.027
2.168	3.066	5.322	29.042	0.058
3.192	4.515	9.113	29.067	0.127
4.700	6.651	14.697	29.122	0.277
6.920	9.804	22.925	29.242	0.603
10.188	14.467	35.061	29.515	1.319
15.000	21.413	53.002	30.163	2.919
Common ratio $q=1{.472,\;q=15}^{1\;/\;n-1}$				

**Table 3:** Experimental condition.

Items	Model or Parameter
Industrial robot—Figure 6a	BRTIRUS0805A (BORUNTE, Guangzhou, China)
Experimental workpiece—Figure 6b	Aluminium round bar (Dongsheng, Guangzhou, China)
Bonnet polishing head—Figure 6c	EVA (PGM, Guangzhou, China)
Lens—Figure 6d	VHX-Z50L (KEYENCE, Osaka, Japan)
Current Source—Figure 6e	Array 3645A (ARRAY, Guangzhou, China)
Polishing fluid—Figure 6f	BT-01 Cerium oxide (Guidechem, Dezhou, China)
Detection Instruments—Figure 6g	VHX-5000 (KEYENCE, Osaka, Japan)

**Table 4:** Trajectory location and bonnet pressing depth setting.

Trajectory Location (mm)	Single-Point Dwell Time (s)	Pressing Depth (mm)
0.050	290.250	0.001
0.331	290.385	0.005
0.885	290.898	0.019
1.976	292.916	0.073
4.125	301.630	0.292
6.977	301.630	0.292
9.828	301.630	0.292
12.680	301.630	0.292
15.531	301.630	0.292
18.382	301.630	0.292
20.000	301.630	0.292

**Table 5:** Comparison of edge contouring results.

	Modified Offset Trajectory	Conventional Grating Trajectory
**Edge collapse amount before polishing (μm)**	347.23	347.23
**Edge collapse amount after polishing (μm)**	345.93	445.90
**Collapsed edge variation (μm)**	1.30 (Decrease)	98.67 (Increase)

**Table 6:** Non-parallel class cut into residual area.

Non-Parallel Tangent Angle α (rad)	Individual Edge Residual Area *S*_1_ (mm^2^)	Total Edge Residual Area *S* (mm^2^)
π/6	2.429	60.725
π/4	1.257	44.450
π/3	0.738	32.110
π/2	0.429	21.450
Trajectory length is 100 mm; radius of polishing area *r* = 1 mm		

**Table 7:** Parallel class cut into residual area.

Parallel Category *d* (mm)	Individual Edge Residual Area *S*_1_ (mm^2^)	Total Edge Residual Area *S* (mm^2^)
0.4*r*	0.003	0.670
0.8*r*	0.022	2.730
1.2*r*	0.077	6.350
1.6*r*	0.193	12.040
2*r*	0.429	21.450
Trajectory length is 100 mm; radius of polishing area *r* = 1 mm		

**Table 8:** Simulation experimental results of the arithmetic progression decreasing rule.

Radius of Polishing Area *R*_1_ (mm)	Residual Height Spacing *d* (mm)	Centre Offset Position *p* (mm)	Dwell Time *t* (s)	Pressing Depth *h* (mm)
1.875	2.652	1.875	29.036	0.044
3.750	5.306	5.854	29.085	0.176
5.625	7.964	12.489	29.166	0.397
7.500	10.630	21.787	29.282	0.709
9.375	13.305	33.754	29.436	1.114
11.250	15.991	48.403	29.631	1.614
13.125	18.693	65.745	29.871	2.214
15.000	21.413	85.798	30.163	2.919

## Data Availability

The data supporting the reported results by the authors can be sent by e-mail.
